# Utilization of tannery wastewater for biofuel production: New insights on microalgae growth and biomass production

**DOI:** 10.1038/s41598-019-57120-4

**Published:** 2020-01-30

**Authors:** Mostafa Nagi, Meilin He, Dan Li, Temesgen Gebreluel, Bian Cheng, Changhai Wang

**Affiliations:** Jiangsu Provincial Key Laboratory of Marine Biology, College of Resources and Environmental Sciences, Nanjing Agricultural University, Nanjing, 210095 China

**Keywords:** Biological techniques, Microbiology, Environmental sciences

## Abstract

Microalgae cultivation on tannery wastewater (TWW) has been examined in some studies as a possible biological application to reduce contamination load and discharge effluents safely. However, Growth aspects, different tolerate strains and enriching the medium were not well investigated. In our study we applied *Scenedesmus* sp., *Chlorella variabilis* and *Chlorella sorokiniana* with different TWW concentrations. *C. sorokiniana* and *C. variabilis* cell density, chlorophyll, and sugar content grew substantially as compared to control. *C.* *sorokiniana* biomass and total lipids folded three and two times in 25% and 40% TWW, respectively as compared to control. *Scenedesmus* sp. showed longer lag phase and lower performance compared to the other two strains. Kelp waste extract (KWE) was added to balance the nutrients supply for *C. sorokiniana*, of which growth and effluents indicators were then greatly promoted in all concentrations. As the lag phase was shortened from 8 to 4 days in 60% concentration, subsequently, chlorophyll, carbohydrates, biomass and total lipids appreciated by 184%, 400%, 162% and 135%, respectively. Furthermore, the COD and ammonium removals improved by 51% and 45%, respectively. These outcomes emphasize the suitability of using TWW for microalgae cultivation with the suitable concentration while adding kelp waste extract for further enhancement.

## Introduction

Tanning Industry is considered to be a major source of pollution and TWW in particular, is a potential environmental concern^1^. Tanning industry wastes poses serious environmental impact on water (with its high oxygen demand, discolouration and toxic chemical constituents^2^), terrestrial and atmospheric systems. Tannery wastewater characteristically contains a complex mixture of both organic and inorganic pollutants. The organic pollutants, such as proteins, carbohydrates, and lipids, are produced from hides washing, while the inorganic pollutants, such as solvents, additives, and chromium, comes from the chemicals added in different treatment processes^3^.

Tanning processes include different steps which in turn produce different chemical compounds and risk elements. Each and every compound needs a specific treatment. Some of these compounds require primary treatment at the tannery scale which needs arrangement and reconstruction of many production units. This makes the treatment process complicated and unaffordable in many developing countries. In the mean while the public awareness of the tanning industry environmental threats has put pressure on governments to limit these threats and put regulations on the industry.

Tanning processes or transforming hides to leather consume large amount of fresh water with an average for the whole process of 25–40 m^3^ per ton of hides. However, the amount can vary on a broad ranging from 10 to 100 m^3^, based on the technology used, the end product, and the type and quality of the used animal hides. The processes from beam house to leather contains three stages; pre tanning process which consume the largest ratio of water among other stages with 15–22 m^3^ per ton of animal hides. However, a significant amount of water is still consumed in the tanning and post tanning processes of 1–2 m^3^ and 2–4 m^3^ per ton of animal hides, respectively^3^.

Several techniques have been studied for tannery wastewater treatment; chemical coagulation, photo-degradation, biodegradation, adsorption, ozonation, electrocoagulation and reverse osmosis. There are some drawbacks for some of these techniques like high consumption of energy, the need for a large area of land or high operation and maintenance cost^4,5^.

## Microalgae Cultivation on Wastewater as A Green Technology

The demand for energy resources is exponentially increasing, which cause escalating trends in producing crops such as maize and soya bean for energy feedstock. It creates the current conflict between utilizing limited good quality land and water resources to human food or energy production. Recently, microalgae cultivation for biofuel production is considered as a green technology for the microalgae photosynthetic rate of CO2 fixation which decreases the net deposit of greenhouse gases. Large requirements of fresh water and nutrients turns the microalgae platform to inefficient in terms of cost benefit analysis^6^. Promoting uses for low quality resources as wastewater for producing biofuel will limit this competition, and allocate the high-quality resources for food production.

Microalgae are known for its versatile nature to grow on various wastewater systems. This ability gives a dual purpose for algae cultivation; water purification, and generating biomass to produce biofuels, or fertilizers^7,8^.

### Returns of cultivation microalgae on wastewater

Microalgae degrade the contaminants directly in wastewater through assimilation of the nutrients like nitrogen and phosphate into their cells for its growth, the used nutrients contribute in algae biomass and the wastewater organic and inorganic load is improved^9^. Furthermore, the drop-in chemicals used in flocculation process during wastewater sedimentation stage as microalgae acts as flocculants which enhances sedimentation rate^10^. Adding microalgae to treat wastewater during primary or secondary treatment, support aerobic microorganism by supplying oxygen which replaces mechanical aeration by photosynthetic oxygenation^9^. The microalgae biodegradation techniques and the ability of microalgae to survive under extreme conditions fuel the trendency of research for microalgae applicability in industrial wastewater. In contrast, Wastewater contamination load replace some of the added nutrients when used as a culture for microalgae cultivation.

### Tannery wastewater as a medium of cultivation

The studies that have been done on using TWW to grow microalgae are still limited; emphasis was directed on TWW treatment and effluents quality indicators as COD, TSS, different nitrogen forms, phosphorous, sulfur, and specific risk elements removal such as chromium, lead, cadmium, Cobber and zinc^11–13^. Limited species of microalgae was used in these studies, Scenedesmus sp. was the most common. TWW as a potential medium was studied in ref. ^4^ where microalgae cultivated in different concentrations of TWW and the effect of the different light intensity on biomass production was investigated. The study used only *Scenedesmus* sp. and determined the optimum concentration and light intensity to maximize biomass and removal ratios. However, the study did not investigate chlorophylls, carbohydrates and lipids productivity.

Up to the authors best of knowledge, different tolerant strains, detailed growth indicators, medium enhancement, and the system limitations have not been investigated thoroughly in the literature. The long acclimation phase due to toxicity effect of high concentrations of ammonium and COD, and lower lipids ratio to biomass are among the main limitations. The possibility to overcome these limitations through system optimization by selecting the tolerate strains and the optimum concentration combined with medium enhancement would place TWW as an option to cultivate microalgae and guide to sustainable application with high efficiency and lower production cost.

The objectives of this study are to investigate the TWW as a potential medium for microalgae cultivation, improve the nutrients balance in the medium, optimize biomass and lipids productivity and lower the time taken to reach the stationary phase. These views would contribute to overcome the limitations of replacing fresh water with low quality water as TWW.

## Materials and Methods

### Tannery wastewater

Tannery wastewater was obtained from a treatment plant for tanneries wastewater located in east of Guannan economic development zone, Lianyungang City, Jiangsu Province, China. The wastewater was collected before treatment and stored frozen at −20 °C until running the experiment. The characteristics of the raw TWW and effluents were analyzed according to the standard methods^14^, as presented in Tables 1 and 3, respectively. Final COD, ammonium and phosphorous were analyzed after microalgae cells harvesting, as presented in Table 3.

**Table 1:** Initial physiochemical characteristics of raw TWW.

Parameter	Value
TN	715 mg.L^−1^
Cl^−^	5960 mg.L^−1^
N-NH_4_^+^	622 mg.L^−1^
P-PO_4_^3−^	6.98 mg.L^−1^
COD	3633 mg.L^−1^
pH	8.3
EC	−68.3 mv

**Table 2:** Comparison of different strains application on different culture conditions and the efficiency in productivity and contamination load removal.

Strains	Culture medium	Light intensity	Nutrients initial mg.L^−1^	Nutrients removal	Biomass	Total lipids %	Ref.
*C. vulgaris*	Primary wastewater + glucose	100	COD: 422.4 N-NH_4_^+^ :28.9 P-Po_4_^3−^:3.2	COD: 67 N-NH_4_^+^:100 P-Po_4_^3−^ :97	0.419	N.A*	^37^
*Scenedesmus abundans* & *C. pyrenoidosa*	rice mill effluent	25.2	COD: 1600 N-NH_4_^+^:154 P-Po_4_^3−^:360	N-NH_4_^+^_:_ 92 & 90.3 P-Po_4_^3−^:98.3 & 97.6	N.A	N.A	^39^
*Scenedesmus* sp.	anaerobic digestion of dairy cattle manure	100	NH_4_^+^ -N:113	N.A	N.A	15.1%	^26^
*Scenedesmus* sp.	biogas and digestated carbon source	149	COD:5000	COD:69.1	1.8	0.7 g.L^−1^	^31^
*Scenedesmus* sp.	60% TWW	80	COD: 2447 N-NH_4_^+^:208 P-Po_4_^3−^:3.96	COD: 57.46 N-NH_4_^+^:60.5 P-Po_4_^3−^:89.50	0.6	N.A	^4^
*C. vulgaris*	treated urban wastewater	143	N-NH_4_^+^:226 P-Po_4_^3−^:143.5	N-NH_4_^+^: 26.5	1.2	N.A	^35^
*Scenedesmus* sp.	50% TWW		P-Po_4_^3–^P:13.23	P-Po_4_^3−^:99.85	0.6	N.A	^13^
*C. minutissima & Scenedesmus* sp.	primary treated wastewater	Solar radiation	COD: 149.75 N-N-NH_4_^+^:39.5 P-Po_4_^3−^: 3.68	COD: 81 N-NH_4_^+^:88.5 &92 P-Po_4_^3−^:85	0.45 & 0.44	11.33 & 81.23	^22^
*C. sorokiniana*	palm oil mill effluent	200	COD: 580 N-NH_4_^+^ :85.8 P-Po_4_^3−^: 20.5	N-NH_4_^+^:93.36 P-Po_4_^3−^ :94.50	1.2	N.A	^24^
*C. sorokiniana* & *Scenedesmus* sp.	100% raw sewage	80	COD: 320 N-NH_4_^+^:52.23 P-Po_4_^3−^:8.47	COD: 69.38, 76.13 N-NH_4_^+^:86.93 &98.54 P-Po_4_^3−^ :68.24 & 98	1.31 & 1.06	27.68% & 28.36%	^25^
*Scenedesmus* sp.	starch containing textile	N.A	COD: 3800	COD:71	1.4	N.A	^30^
*Acutodesmus obliquus* & *Parachlorella kessleri*	10% anaerobic digestion effluent	200	N.A	COD: 45.4, 39.1 N-NH_4_^+^:7.87 & 15.3 P-Po_4_^3−^:84 & 84.2	1.1 & 1	84.2 & 84	^21^
*Scenedesmus* sp.	molasses and grass wastewater anaerobic digested effluents	300	N-NH_4_^+^:159 P-Po_4_^3−^:16.71	N.A	3.2	34%	^33^
*Scenedesmus obliquus* & *Desmodesmus* spp.	land fill leachate and urban wastewater	53	COD: 465 N- NH_4_^+^:150 P-Po_4_^3−^: 20	COD: 64 & 67 N-NH_4_^+^: 79 & 82 P-Po_4_^3−^:43 & 41	1.2 &1.3	N.A	^27^

*N.A = Not available in the literature.

### Experiment design

Three strains of microalgae, *C. sorokiniana* (FACHB-275), *Scenedesmus* sp. (FACHB-489) and *C. variabillis* (FACHB-171) were used in this experiment and were provided by Institute of Hydrobiology, Chinese Academy of Sciences. One-liter of Erlenmeyer flasks with 500 ml working volume was used. Each strain was incubated for 14 days before use as inoculum and the experiment was running for 16 days. Temperature adapted at 25 °C with shaking twice a day and continuous lighting of 40 µmol photons m^−2^.S^−1^ and a 14/10 h light/dark cycle.

The medium used for the seed culture and as a control was BG11 for both *C. sorokiniana* and *Scenedesmus* sp. and contains the following: 1.5 g NaNO_3_, 40 mg K_2_HPO_4_, 75 mg MgSO_4_·7H_2_O, 36 mg CaCl_2_·2H_2_O, 20 mg Na_2_CO_3_, 0.006 mg Fe(NH_4_)_3_(C_6_H_5_O_7_)_2_, 0.001 mg Na_2_EDTA, 0.006 mg citric acid and 1 mL of A5 solution^15^ in 1 L of fresh water. *C. variabilis* was grown in the modified Zarrouk’s medium (NaNO_3_: 2.50 g.L^−1^, K_2_HPO_4_: 0.50 g.L^−1^, NaHCO_3_: 16.80 g.L^−1^, NaCl: 1.00 g.L^−1^, MgSO_4_·7H_2_O: 0.2 g.L^−1^,CaCl_2_·2H_2_O: 0.02 g.L^−1^, FeSO_4_· 7H_2_O: 0.01 g.L^−1^) and 1 mL of trace metals solution^16,17^. The inoculum was 16% (v/v) for each strain. The initial cells densities were found to be 6.54 * 10^6^, 7.15 * 10^6^ and 8.68 * 10^6^ cells/ml for *C. sorokiniana*, *Scenedesmus* sp. and *C. variabilis*, respectively. Raw tannery wastewater was diluted with distilled water to three concentrations (25%, 40% and 60%; v/v). We later chose the outperforming tested strain to apply 6% kelp waste extract which contents are presented in Table 4 as reported by ref. ^18^. The optimum percentage reported in the same research was 8%, however, we chose to lower the ratio as TWW has high load of nutrients compared to BBM. We run this treatment under the same concentrations and growth conditions for inoculum and TWW batch experiment. Each treatment was done in triplicate. Other cultivation conditions were the same as for inoculum.

### Determination of algal growth

Samples were taken for the three strains on daily basis to measure sugar, chlorophyll content, optical and cells density, finally, the harvested biomass were assessed gravimetrically. Biomass was harvested using centrifugation at 4 °C, 8000 rpm for 10 min and freeze dried using lyophilizer for further analysis. The total lipids were extracted from the harvested biomass using solvent extraction; chloroform and methanol (2:1 v/v) based on a modified method^8^. The mixture sonicated for 10 min in an ice bath for cell disruption.

### Analysis of intracellular sugar

A 5 mL sample was collected and centrifuged at 8000 rpm for 10 min. Then, 5 ml of distilled water was added to the pellet and stored at −20 °C for 12 h; freeze-thawed, this process was repeated 3 times after which the samples were sonicated for 10 min in an ice bath. After that, the samples were centrifuged. One mL of supernatant was mixed with 0.5 mL phenol solution (6.0%, v/v) and 2.5 mL concentrated sulfuric acid and incubated at 37 °C for 30 min. The results were recorded spectrophotometrically at 490 nm. The phenol, sulphuric acid method^19^.

### Measurement of cells density and pigments

To evaluate the growth of the three strains, chlorophyll and optical density measurements were conducted on daily basis. The content of chlorophyll (a + b) was determined using a modified methanol method^20^. 5 mL culture was sampled and centrifuged at 8000 rpm for 10 min. The pellets were extracted by 5 mL methanol at 4 °C for 24 h in darkness. The absorbance of extracts at 653 and 666 nm was analyzed with SpectraMax M5 Microplate Reader (Molecular Device, USA). Optical density was measured at OD750. Optical density was interrelated with cells number to verify the relation. The number of cells was counted using a hemacytometer under an optical microscope (Nikon YS 100, Japan).

Correlation was found as follows:

y = 0.0722x − 8.4532 with R² = 0.9801 for *C. sorokiniana*.

y = 0.0657x − 2.5701 with R² = 0.9915 for *Scenedesmus* sp.

y = 0.0697x − 0.267 with R² = 0.9945 for *C. variabilis*

### Statistical analysis

The error bars indicate the standard deviation among the replicates. Measurements were carried out in triplicate and the mean values of the triplicates were plotted. Comparisons of means were conducted by one-way analysis of variance (ANOVA), followed by Bonferroni tests to identify the sources of detected significance. In all cases, comparisons that showed a p value < 0.05 were considered significant.

## Results and Discussion

### Cells density and general growth performance

**Figure 1:**
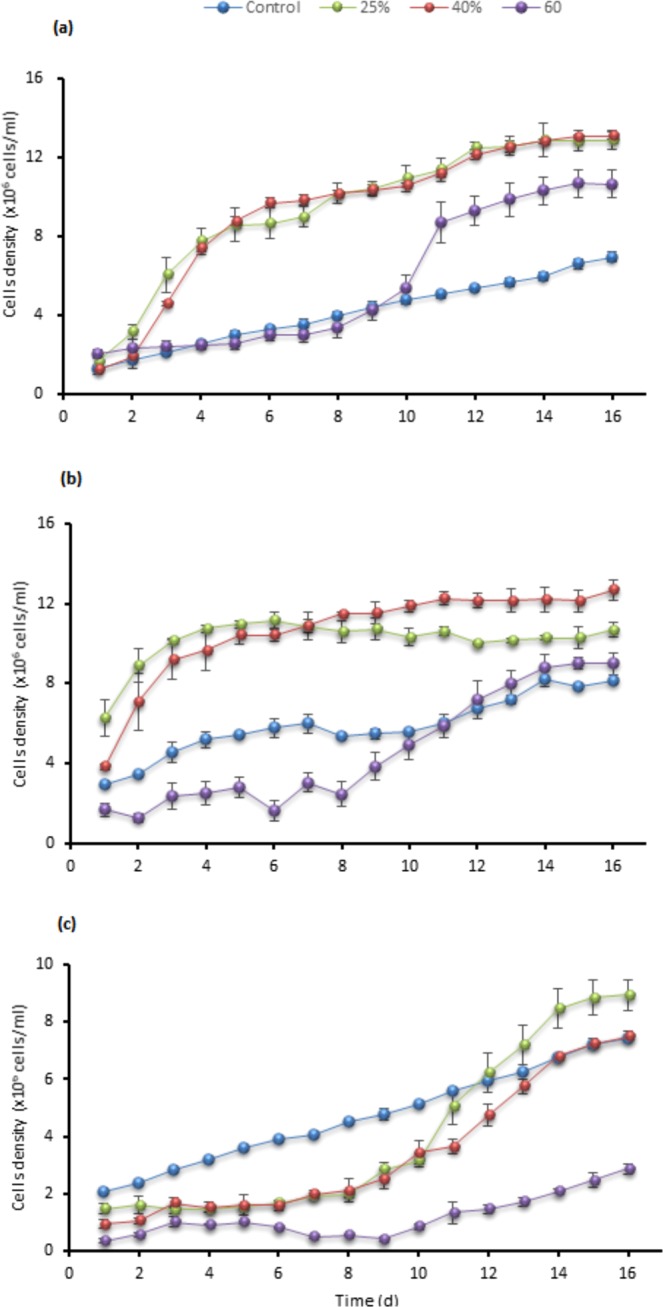
Cells density growth trends in (**a**) *C. sorokiniana* (**b**) *C. variabilis* and (**c**) *Scenedesmus* sp. under different TWW concentrations (25%, 40%, and 60%). All plotted data were mean ± standard deviation of n = 3.

**Figure 2:**
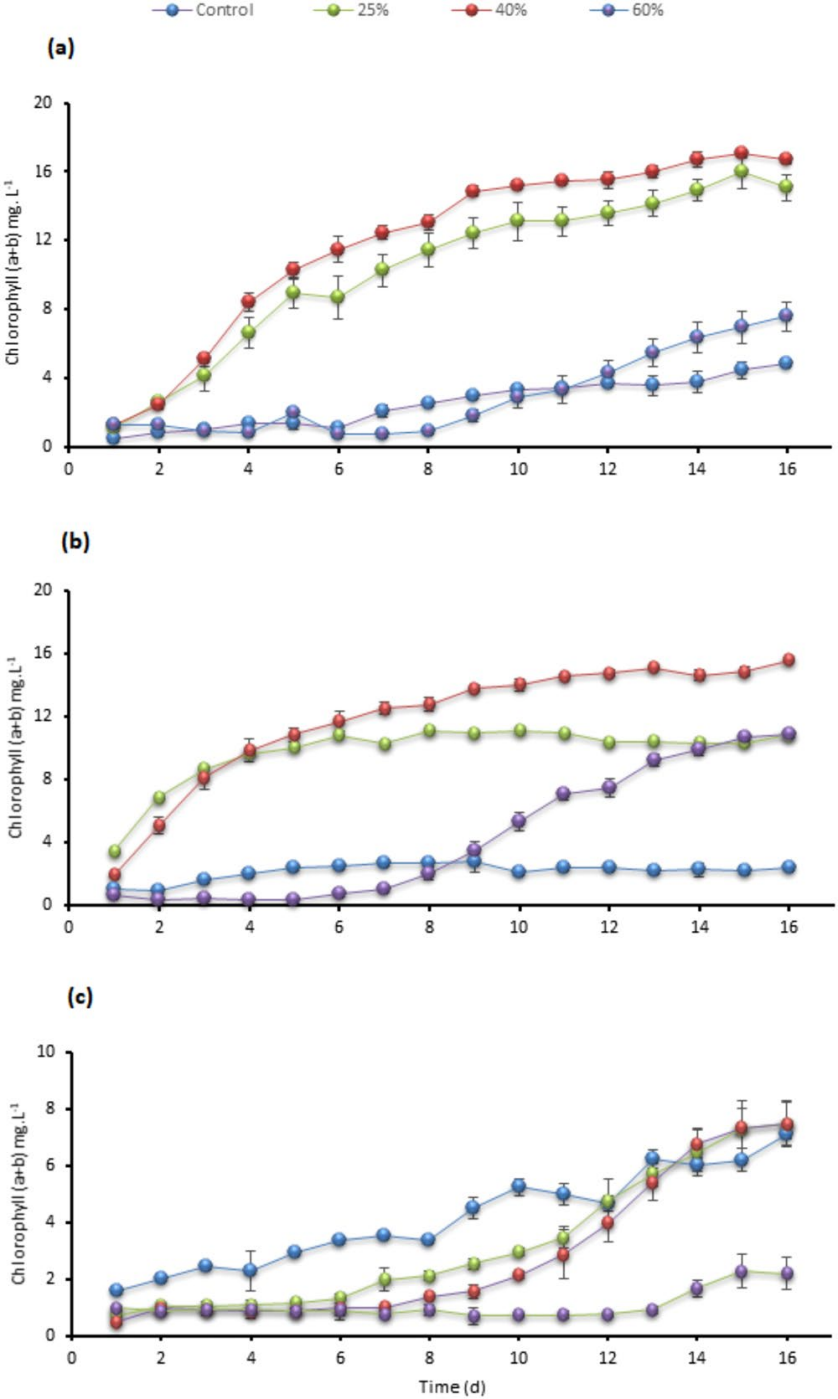
Comparison of the changes in chlorophyll (a + b) contents in (**a**) *C. sorokiniana* (**b**) *C. variabilis*, and (**c**) *Scenedesmus* sp. under different TWW concentrations (25%, 40%, and 60%) presented in mg. L^−1^. All plotted data were mean ± standard deviation of n = 3.

**Figure 3:**
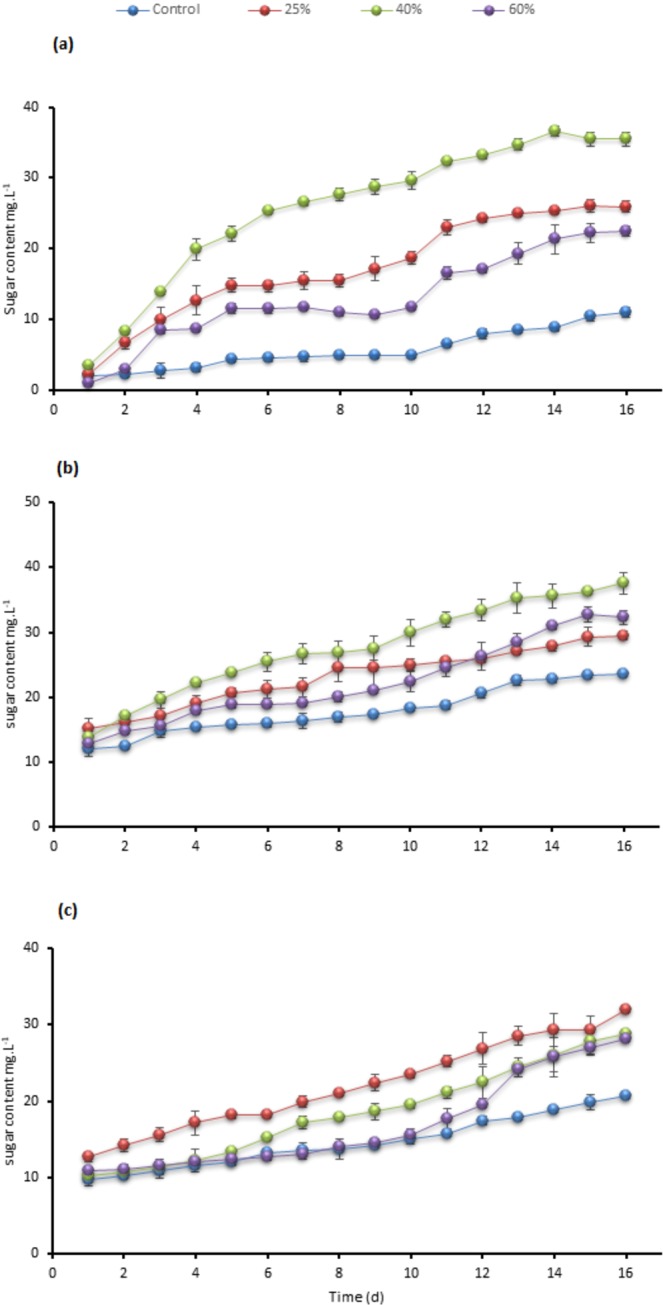
Comparison of the changes in intracellular soluble sugar contents in (**a**) *C. sorokiniana* (**b**) *C. variabilis* and (c) *Scenedesmus* sp. under different TWW concentrations. The sugar content is presented as mg. L^−1^. All plotted data were mean ± standard deviation of n = 3.

**Figure 4:**
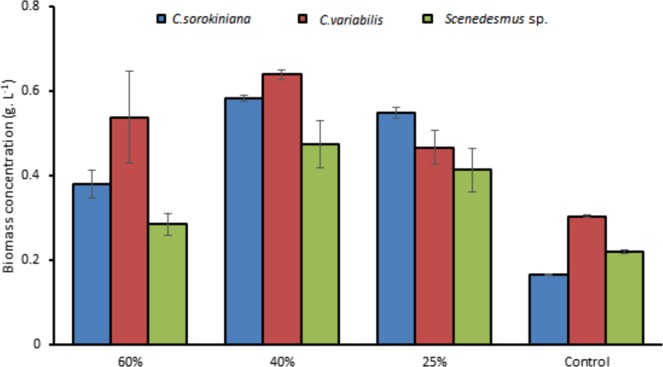
Dry cell weight for (*C. sorokiniana*, *C. variabilis* and *Scenedesmus* sp.) in different TWW concentrations presented in g.L^−1^. All plotted data were mean ± standard deviation of n = 3.

Substantial growth and tolerance for the three tested strains at 40% TWW were observed. Testing at 40% concentrations showed the maximum cells density, chlorophyll, intracellular sugar, and biomass as presented in Figs. 1, 2, 3 and 4 in *C. sorokiniana* and *C. variabilis* followed by 25%. However, 25% exceeded the performance of 40% in *Scenedesmus* sp. in chlorophyll, intracellular sugar, cells density, biomass and total lipids. The acceleration phase started directly after incubation in 25% and 40% concentrations. The quick response and adaptation for these concentrations is possibly initiated from the richness of tannery wastewater with nitrogen and organic content and acceptable load for the tested strain. At 60% concentration, a slow or no growth was observed during the first week and was lower in growth parameters than control at the start of the second week. While at the last days of the experiment it recorded high growth rate to surpass the control in biomass, chlorophyll, cells density, Intracellular sugar in *C. sorokiniana* and *C. variabilis*. Furthermore, in *Scenedesmus* sp. 60% records the lowest chlorophyll and cells density in *Scenedesmus* sp.

TWW at 60% concentration inhibited the growth of microalgae cells and extended lag phase (phase of adaptation of microalgae to tannery wastewater) for 7 days in both *C. sorokiniana* and *C. variabilis* and 13 days for *Scenedesmus* sp. Results indicate that this higher load of nitrogen, organic matter, and risk elements could be unsuitable for microalgae growth, consequently, microalgae cells need more time to acclimate to the conditions in the medium. Results also indicate that over 60% could be toxic for cells and may cause total suspension of the growth. Same direction discussed in ref. ^21^ as 10% anaerobic digestion effluent inhibits the growth for 9 to 12 days when *Parachlorella kessleri* and *Acutodesmus obliquus were* applied, however, both strains gained 1.1 g.L^−1^ and 1 g.L^−1^ of biomass, respectively after 25 days (Table 2).

### Chlorophylls and carbohydrates

The case of 40% concentration in *C. sorokiniana* showed a higher chlorophyll (a + b) content than 25% since third day and ends with 16.71 mg.L^−1^ (Fig. 2a). Additionally, 60% showed a lower chlorophyll content than control till the twelfth day to end with 7.58 mg.L^−1^. *C. variabilis* has the same state as *C. sorokiniana*, 40% surpassed 25% at the fifth day and recorded the highest chlorophyll content with 15.55 mg.L^−1^ (Fig. 2b), while 60% chlorophyll content exceeded the control at 9th day to end with 10.81 mg.L^−1^. *Scenedesmus* sp. had different growth trends than both *C. sorokiniana* and *C. variabilis*. As presented in Fig. 2c, 25% and 40% concentrations ends with similar content 7.45 mg.L^−1^ slightly higher than control 7.07 mg.L^−1^. A little increase in chlorophyll content at 60% TWW viewed at the last three days before harvesting to end up with 2.19 mg.L^−1^.

In chlorophyll graphs, 40% and 25% reached to stationary phase in *C. sorokiniana* and *C. variabilis* strains at the 8th–10th day, however, 60% spent longer time to reach the same phase. In *Scenedesmus* sp. 25% and 40% remained longer time at lag phase which delayed stationary phase and both concentrations were lower in performance compared to control until day 12. *Scenedesmus* sp. and C*hlorella minutissima* were applied on primary treated wastewater^22^. Chlorophyll content recorded the maximum after 10 days with 18 mg.L^−1^ and 12 mg.L^−1^, respectively. Chlorophyll for *Scenedesmus abundans* and *Chlorella pyrenoidosa* cultivated on rice mill effluent achieved 3.88 mg.L^−1^ and 5.55 mg.L^−1^, respectively^1^.

Intracellular sugar in *C. sorokiniana* displayed similarity in trends with chlorophyll (a + b) content as shown in Figs. 2a and 3a. The concentration of 40% gained the highest sugar content of 35.44 mg.L^−1^ compared to 25.93 mg.L^−1^, 22.44 mg.L^−1^ and 11.02 mg.L^−1^ for 25%, 60% and control, respectively. *Chlorella variabilis* intracellular sugar content (Fig. 3b) presented greater content for 60% concentration over 25% with 32.29 mg.L^−1^, 29.48 mg.L^−1^ sugar content, respectively. Sugar content of 25% in *Scenedesmus* sp. was 31.89 mg.L^−1^ while 40% and 60% sugar content were similar before harvesting (Fig. 3c). Five species of *C. sorokiniana*, *chlorella vulgaris* and *Scenedesmus* sp. were cultivated on primary and secondary treated wastewater combined with anaerobic digestion centrate. Initial COD and ammonium were 590 mg.L^−1^ and 176 mg.L^−1^, respectively^23^. Different inoculum volumes and different light intensities were applied; outcomes were in the same direction (Table 2).

### Biomass and lipids content

Biomass was highest in *Chlorella variabilis* 40% TWW concentration with 0.64 g.L^−1^ followed by 0.58 g.L^−1^, 0.48 g.L^−1^ for *C. sorokiniana* and *Scenedesmus* sp., respectively (Fig. 4). *C. sorokiniana* demonstrated the highest biomass in 25% with 0.55 g.L^−1^ followed by 0.47 g.L^−1^, 0.41 g.L^−1^ for *C. variabilis* and *Scenedesmus* sp., respectively. In both *Scenedesmus* sp. and *C. sorokiniana* 60% TWW presented lower biomass than the other two concentrations, however, *C. variabilis* 60% biomass was not statically significant to 25% concentration. The Reported *Scenedesmus* sp. biomass was 0.6 g.L^−1^ after 25 days under aerated conditions and 80 µmol photons m^−2^.S^−1^ light intensity in 60% TWW medium^4^. In ref. ^13^, when *Scenedesmus* sp. applied on 50% of TWW, the biomass was 0.6 g.L^−1^, and on palm oil mill effluent was 1.2 g.L^−1^ ^24^ by *C. sorokiniana* (Table 2).

**Figure 5:**
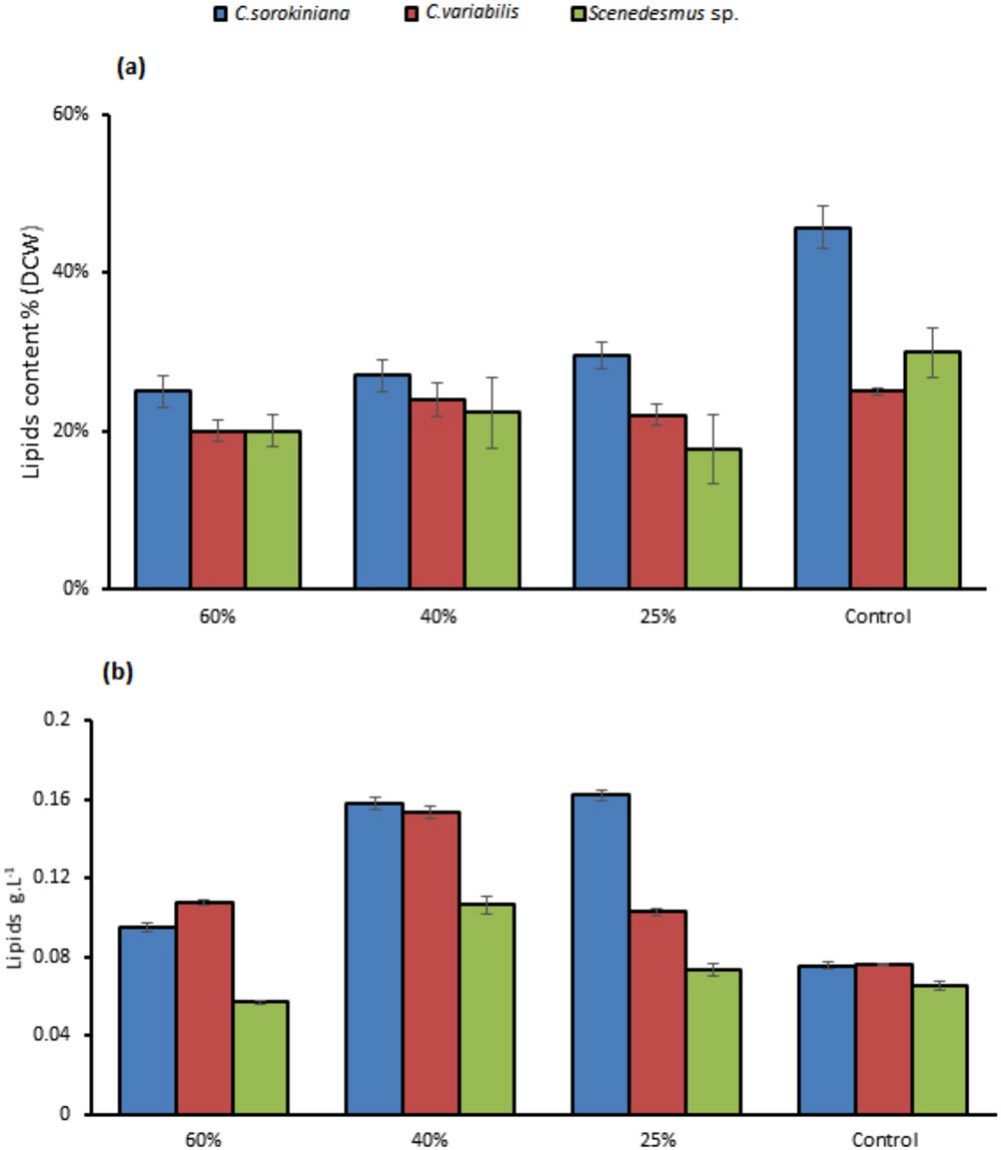
(**a**) Lipids content as % of dry cell weight (**b**) total lipids as g.L^−1^ in (*C. sorokiniana*, *C. variabilis*, and *Scenedesmus* sp.) under different TWW concentrations. All plotted data were mean ± standard deviation of n = 3.

**Figure 6:**
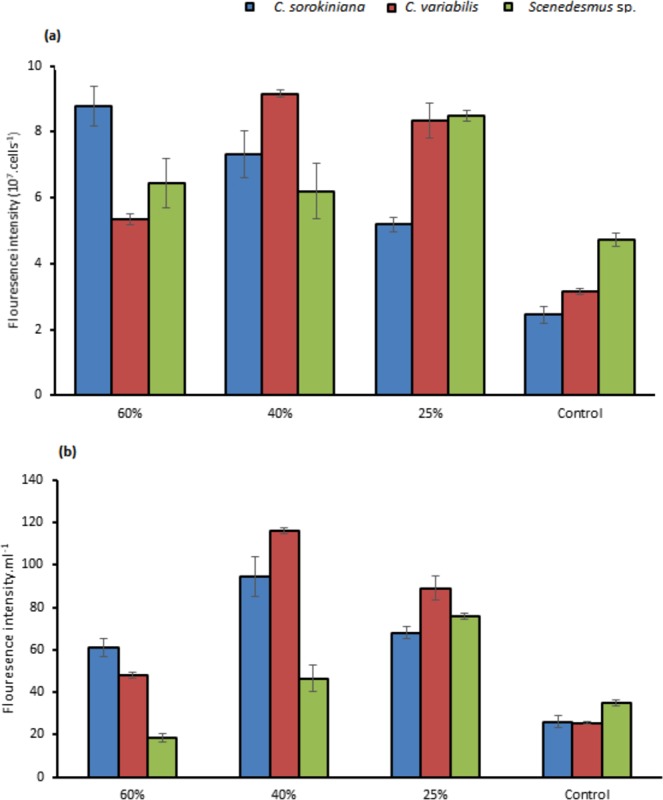
Fluorescence intensity in (*C. sorokiniana*, *C. variabilis* and *Scenedesmus* sp.) under different TWW concentrations presented in (**a**) (10^7^ cells. ml^−1^, (**b**) O.D.ml^−1^. All plotted data were mean ± standard deviation of n = 3.

*C. sorokiniana* total lipids for 25% and 40% TWW were not varied statistically with 162 mg.L^−1^, 158 mg.L^−1^, respectively (Fig. 5a,b). *C. variabilis* showed high variation between 40% and 25% in total lipids with 154 mg.L^−1^, and 103 mg.L^−1^ respectively. Lipids content of 60% TWW were 108 mg.L^−1^ and 95 mg.L^−1^ in *C. variabilis* and *C. sorokiniana*, respectively. In *Scenedesmus* sp. the highest total lipids were in 40% concentration with 106 mg.L^−1^. When *C*. *sorokiniana* and *Scenedesmus* sp. applied on different concentrations of raw sewage under 80 µmol photons m^−2^.S^−1^ and 80 rpm continuous shaking, total lipids obtained were 362 mg.L^−1^ and 300 mg.L^−1^ for *C. sorokiniana* and *Scenedesmus* sp., respectively^25^. Total lipids content was 151 g.kg^−1^ dry biomass for *Scenedesmus* sp. with anaerobic digestion of dairy cattle manure^26^. *Scenedesmus* sp. and C*hlorella minutissima* lipids productivity were 11.33 mg.L^−1^ and 81.23 mg.L^−1^, and Biomass production was 0.4 g.L^−1^ ^22^ (Table 2). In our experiment total lipids ratios were the maximum in control medium in the three strains with 46%, 30%, and 25% for *C. sorokiniana*, *C. variabilis*, and *Scenedesmus* sp., respectively (Fig. 5a). In contrast, the control presented the lowest production of lipids content per liter except in *Scenedesmus* sp. (Fig. 5b). Higher lipids content in control as a ratio of biomass were found in ref. ^27^. The authors stated that Stress conditions, nitrogen enrichment and C/N ratio were leading to changes in carbohydrates, protein and lipids fractions. It was observed in ref. ^28^ that when nitrogen is sufficient the protein content and carbohydrates is increasing and lower lipids content are gained. The same direction confirmed in ref. ^29^ where microalgae cultivation on wastewater gained lower lipids ratios. Represented as a fluorescence intensity/cells (Fig. 6a) neutral lipids in all TWW concentrations showed higher content compared to control. As fluorescence intensity/ml 40% TWW recorded the highest in *C. variabilis* and *C. sorokiniana* among the three concentrations and control (Fig. 6b).

### Effluents quality

Efficiency of contamination load removal achieved the maximum in 25%, 40% concentrations in both *C. sorokiniana* and *C. variabilis*, as presented in Table 3. *C. variabilis* COD removals reached 84% and 80% for 40% and 25% concentrations, respectively. Different studies discussed the ability of microalgae to adopt to high COD levels and remove large portions. In ref. ^30^ starch containing textile wastewater COD processed to anaerobic fermenter before *Scenedesmus* sp. cultivation to reduce COD from 39000 mg.L^−1^. Algae cultivation initiated at 3800 mg.L^−1^ COD, and the reported removal ratio was 71.1%. *Scenedesmus* sp. cultivated on TWW contain COD 4000 mg.L^−1^ in ref. ^4^ at the highest concentration, and in ref. ^31^ COD was 5000 mg.L^−1^. Tolerance to high COD varies based on different factors which are mentioned in more details in next section. Phosphorous removal achieved 93% in *C. sorokiniana* for 40% and 25% concentrations. Ammonium removal was the highest in *C. sorokiniana* 25% concentration with 74% and the second highest at *C. variabilis* 25% with 68%. *Scenedesmus* sp. removal ratios were lower compared to the other two strains in all concentrations. *Scenedesmus* sp. also presented the lowest removal in all treatments in 60% TWW with 55%, 38%, and 36% in phosphorous, COD, and ammonium, respectively. Both concentrations 25% and 40% displayed higher removal ratios compared to TWW 60% concentration. The initial contents are much higher in 60% concentration, in addition to the longer growth acclimation phase for microalgae cells in 60% concentration which contributed to the lower removal ratios. *C. sorokiniana* and *C. variabilis* demonstrated higher removal ratios than *Scenedesmus* sp. It is consistent with the growth parameters results as both strains showed high content of biomass, total lipids and growth rates than *Scenedesmus* sp. In ref. ^24^ inoculum ratio was 28% of *C. sorokiniana* added to palm mill effluent, and 93.36% of NH_4_^+^ and 94.50% of PO_4_^3−^ were successfully removed. The results indicated that biomass increased with the increase of N from 221 to 645 mg.L^−1^ and the increase of P from 5 to 15 mg.L^−1^. *C. sorokiniana* has achieved the highest removal efficacy in aquaculture wastewater among *Scenedesmus obliquus* and *Ankistrodesmus falcatus*^7^. In ref. ^4^ *Scenedesmus* sp. removed 57.46%, 60.5% and 89.5% of COD, ammonium and phosphorous respectively. *Scenedesmus* sp. and *C. minutissima* COD removals were 80% for both strains while ammonium and phosphorous removals were 90% ± 2 and 70% ± 2, respectively^22^ (Table 2).

**Table 3:** Final effluents and removal ratios of COD, N-NH_4_^+^, and P-PO_4_^3−^ after biomass harvesting in (a) *C. sorokiniana* (b) *C. variabilis*, and (c) *Scenedesmus* sp. under different TWW concentrations (25%, 40%, and 60%).

	TWW	COD		N-NH_4_^+^		P-PO_4_^3−^	
		Effluent	Removal %	Effluent	Removal%	Effluent	Removal%
*C. Sorokiniana*	25%	181	80 ± 1.32	37.34	74 ± 3.28	0.122	93 ± 2.55
	40%	377	74 ± 3.38	109.53	56 ± 1.21	0.196	93 ± 2.51
	60%	1046	52 ± 4.51	194.17	48 ± 1.04	0.46	89 ± 2.43
*C. Variabilis*	25%	145	84 ± 1.42	49.78	68 ± 2.64	0.122	93 ± 2.88
	40%	288	80 ± 1.35	93.82	62 ± 1.90	0.37	87 ± 2.03
	60%	777	64 ± 2.87	213.14	43 ± 5.76	0.94	78 ± 3.25
*Scenedesmus* sp.	25%	308	66 ± 2.39	82.26	47 ± 0.13	0.53	70 ± 2.21
	40%	642	56 ± 4.02	152.61	39 ± 1.83	1.01	64 ± 3.52
	60%	1351	38 ± 1.15	239.27	36 ± 0.95	1.9	55 ± 0.76

The results are presented by mean ± SD, n = 3, effluents are in mg.L^−1^.

### Growth inhibition

Adaptation of microalgae cells to tannery wastewater can represent a challenge for the system as cells spends time in lag phase which affects production cycle and the economics of the scale. Lag phase can extend for long time when the strain has low tolerance to the high load of some nutrients and risk elements in tannery wastewater especially when higher concentration is made such as 60% TWW. Our results indicate that high concentration (60% or over) could cause longer lag phase. It is conceivably driven by high load of risk elements and other nutrients. The slow growth and the decrease in cells number that *Scenedesmus* sp. experience in tannery wastewater in the first 2–3 days discussed in other studies^4,11^. In our experiment conditions *C. sorokiniana* and *C. variabilis* displayed strong growth from the first day on 25% and 40% TWW concentration. Concentrations of 60% TWW showed relatively low performance and no significant growth in the three strains during the first week; however, the 8^th^ day witnessed initial of growth for *C. sorokiniana* and *C. variabilis*. In contrast, *Scenedesmus* sp. low performance state continued till day13^th^. In ref. ^11^ TWW concentration over 75% level off the growth after six days in *Scenedesmus* sp. The optimum growth was found to occur in 50% concentration under 54 µmol photons m^−2^. S^−1^ light intensity.

As different sources of wastewater are characterized by high nitrogen and COD content, similar growth curves to our results were discussed in ref. ^26^, where dairy cattle digested manure used as a medium for *Scenedesmus* sp. Microalgae cells were growing rapidly at the first 2 to 3 days due to the high content of nitrogen, however, the growth rate diminished later. It was concluded that the higher the ammonium concentration, the higher the continuity of the growth rate till certain level, the authors stated that over 10% digestate inhibit the growth of cells with ammonium concentration above 113 mg.L^−1^. In another study^32^ the tolerance limit of *Scenedesmus* sp. was 100 mg.L^−1^ and higher concentration would be toxic and could inhibit the cells growth.

Anaerobic digested effluent of grass and molasses were applied as a medium for two strains of *Scenedesmus* sp. and initial ammonium was 159 mg.L^−1^ ^33^. *Scenedesmus* sp. was applied for 16 days at 300 µmol photons m^−2^ s^−1^ light intensity with continues aeration 1–2% CO_2_ and gained 3.2 g.L^−1^ of biomass, 7 * 10^−7^ cells.ml^−1^ and the total lipids and carbohydrates content were 34% and 30%, respectively. In another study^4^ *Scenedesmus* sp. cultivated on TWW concentration 20–100% with different light intensity; 80–200 µmol photons m^−2^ s^−1^, and continues aeration for 24 days was applied. Results indicated the ability of removal for high load of TN, ammonium and phosphorous. The authors did not observe stationary or decay phases and they proposed that the lag phase longer duration delayed the stationery and decay phases.

*C. sorokiniana* presented the highest ammonium removal among *C. vulgaris*, *Scenedesmus obliquus* and *Selenastrum capricornutum*^34^. The study reported that ammonium concentration tolerance limit is above 210 mg.L^−1^ which completely inhibits *C. sorokiniana* growth. In ref. ^35^ 1.2 g.L^−1^ of dry biomass harvested when *C. Vulgaris* applied on high ammonium concentration (226 mg.L^−1^) added to treated urban wastewater under continues CO_2_ aeration conditions and 143 µmol photons m^−2^.S^−1^.

In our study, unlike *C. sorokiniana* and *C. variabilis*, *Scenedesmus* sp. spent longer time for acclimation for all concentrations with less growth performance which indicate the lower tolerance of *Scenedesmus* sp. comparing to the other two strains.

In the light of the presented previous studies, including our results, we could find variation in tolerated limits, acclimation periods, growth parameters, and nutrients removal. Several external factors related to the cultivation conditions could contribute to this variation such as Inoculum size, light intensity and continues aeration which has a major effect on microalgae tolerance to extreme conditions in the medium^23,24^.

### KWE supplement

**Figure 7:**
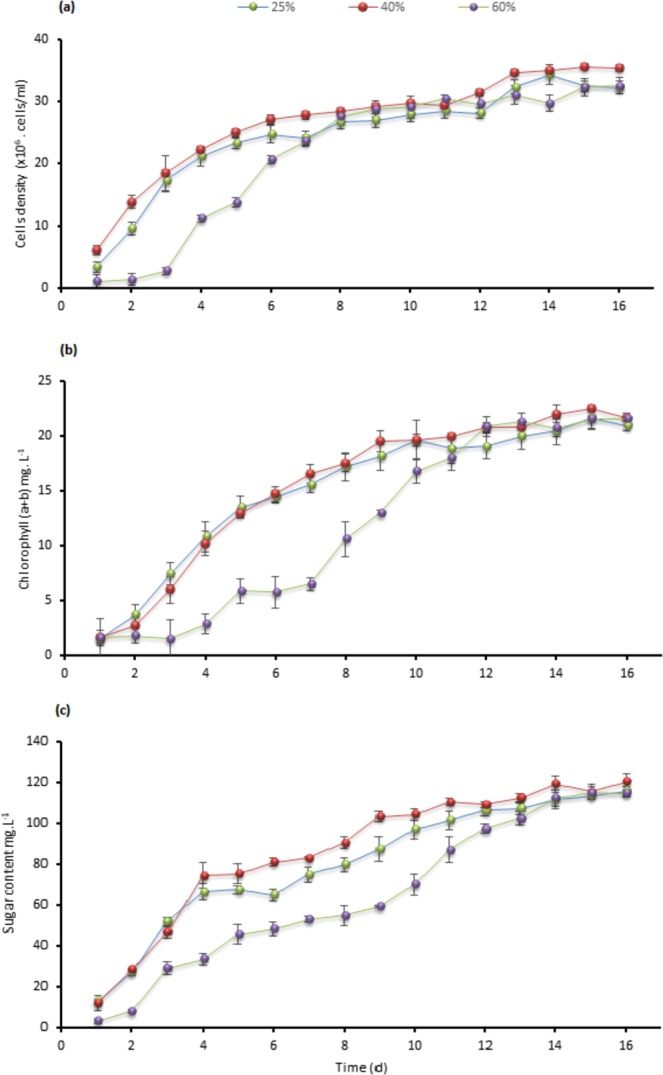
(**a**) Cells density (**b**) chlorophyll (a + b) (**c**) intracellular sugar growth trends in *C. sorokiniana*, TWW concentrations (60%, 40%, and 25%) combined with KWE 6%. All plotted data were mean ± standard deviation of n = 3.

*C. sorokiniana* cultivated in modified TWW with KWE showed a higher growth indicated by chlorophyll, cell density, intracellular sugar, biomass and total lipids. Substantial growth enhancement was observed for all concentrations under KWE supplement, while the differences in cell density and chlorophyll were not considerable among the three concentrations as presented in Fig. 7a,b. The highest change among the three concentrations occurred at 60% TWW with KWE compared to 60% TWW unaccompanied which indicates better acclimation and tolerance optimizing. Lag phase continued for shorter time in 60% concentration comparing to TWW alone. Chlorophyll reached 21.55 mg.L^−1^ at the 16th day in 40% concentration (Fig. 7b). The chlorophyll reported in our study is distinguished to the reported in ref. ^18^ as TWW replaced BG11 medium. It is consistent with the findings mentioned in another section regarding nitrogen rich medium^27–29^. Intracellular sugar presented high content in TWW with KWE compared to TWW without modification. Sugar content reached 120.2 mg.L^−1^ in 40% TWW and other two concentrations were in the same range (Fig. 7c). Results consistant with ref. ^18^, however, adding higher KWE in that study lead to higher intracellular sugar.

**Table 4:** KWE contents^15^.

Parameter	Value
N (mg. L^−1^)	5723.93 ± 75.21
P (mg. L^−1^)	5529.45 ± 33.94
K (mg. L^−1^)	60.54 ± 0.43
Ca (mg. L^−1^)	54.91 ± 4.51
Mg (mg. L^−1^)	75.64 ± 5.94
Fe (mg. L^−1^)	ND
Mn (mg. L^−1^)	0.65 ± 0.06
Cu (mg. L^−1^)	0.04 ± 0.09
Zn (mg. L^−1^)	8.3 ± 1.75
B (mg. L^−1^)	6.04 ± 0.85
Amino acids (mg. L^−1^)	194.03 ± 0.75
Reducing sugars (g.L^−1^)	19.55 ± 0.13
Total sugars (g.L^−1^)	23.19 ± 0.65
Alginic acid (g.L^−1^)	6.09 ± 0.44

**Figure 8:**
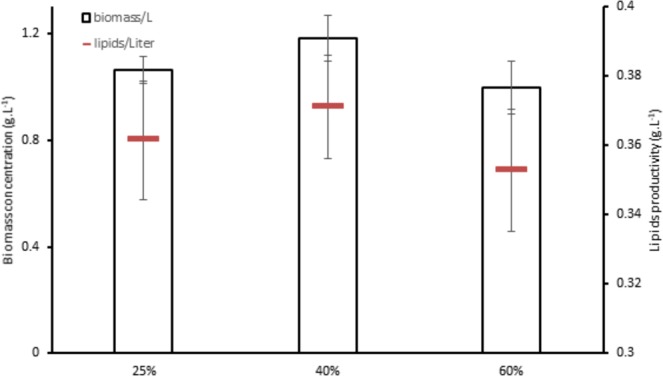
*C. sorokiniana* biomass and lipids productivity in different TWW concentrations with 6% KWE. All plotted data were mean ± standard deviation of n = 3.

**Figure 9:**
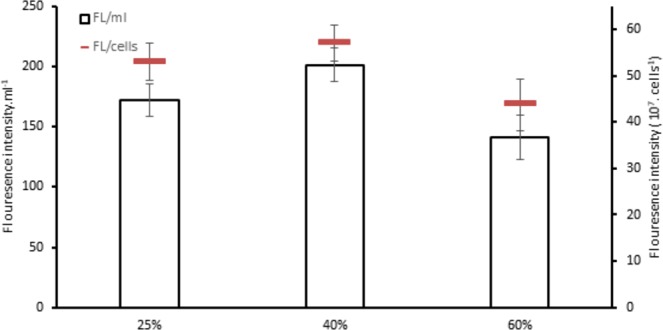
*C. sorokiniana* fluorescence intensity in TWW different concentrations modified with 6% KWE. All plotted data were mean ± standard deviation of n = 3.

Application of 6% KWE demonstrated great enhancement in biomass as presented in Fig. 8, biomass reached 1.18 g.L^−1^ in 40% concentration; and 1.06 g.L^−1^ and 0.997 g.L^−1^ for 25% and 60%, respectively. The Cells density in our study were comparable with ref. ^36^, while the biomass of microalgae was 0.727 g.L^−1^ in the same reference. In ref. ^37^ *C. vulgaris* cells density was stable around 2 * 10^7^ cells.ml^−1^ on primary wastewater unaccompanied, however, when glucose added the cells density reached 6 * 10^7^ cells.ml^−1^ at the 5^th^ day. Furthermore, the total carbohydrates was less than 10 mg.l^−1^ and as a result of the treatment it increased by 300%. *Scenedesmus* sp. cultivated on biogas and digestated carbon source reported to produce 1.8 g.L^−1^ of dry biomass^31^. A slight change in total lipids in percentage (Fig. 8), but the total accumulated content was considerably greater than TWW alone. Total content reached 0.371 g.L^−1^ in 40% TWW concentration. Neutral lipids content witnessed enhancement in all concentrations compared to *C. sorokiniana* applied in TWW alone as presented in Fig. 9. The increase in 25% neutral lipids reached 2.5 times the TWW without KWE supplementation. More than two times increase was observed in 40% and 60% in neutral lipids as well. Total lipids reported in ref. ^18^ were less than 30% as a percentage of biomass at 6% KWE while neutral lipids were similar to our study.

**Figure 10:**
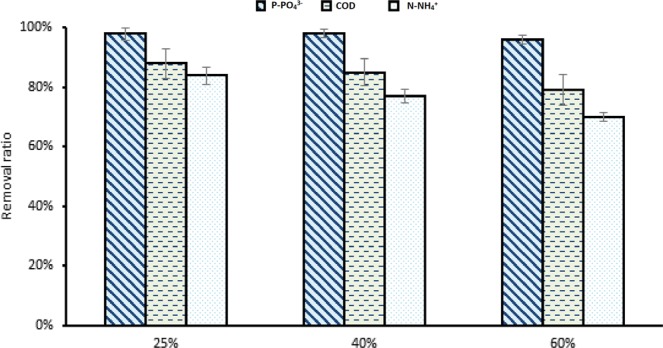
*C. sorokiniana* removal efficiency in TWW different concentrations with 6% KWE. All plotted data were mean ± standard deviation of n = 3.

Water quality parameters were enhanced when KWE was applied to TWW concentrations as presented in Fig. 10. COD, ammonium and phosphorous removal ratios were considerably improved parallelly with growth parameters. COD removal increased to reach 88% followed by 84% and 79% for 25%,40%, and 60%, respectively. *Scenedesmus* sp. on biogas and digestate carbon source, COD removal could reach 69% when Initial COD was 5000 mg.L^−1^ ^31^. Ammonium removal ratios witnessed a substantial change to be 84%, 77%, and 70% for 25%, 40%, and 60% TWW, respectively. Phosphorous removal slightly increased in 25% and 40% TWW to be 98% for both and 96% for 60% TWW concentrations.

As KWE proved to have a significant impact on yield when added to the medium as reported in different studies^18,36^. Adding KWE to TWW to grow *C. sorokiniana* reduced the lag phase period, and the accelerating growth phase started on the 4^th^ day in 60% TWW. As a result, chlorophyll, carbohydrates, biomass and total lipids appreciated by 184%, 400%, 162% and 135%, respectively. Additionally, the COD and ammonium removals improved by 51% and 45%, respectively.

These outcomes indicate that supplying and balancing the nutrients would improve the tolerance of the strain to the extreme conditions in the medium. Adding KWE also improves the nutrients removal in all TWW concentrations and increases all growth parameters. Same trend confirmed in ref. ^37^ when glycerol and glucose added to primary wastewater the removal ratios folded in ammonium and phosphorous.

### TWW as a potential medium for microalgae production

The power intensity demanded by TWW treatment in china was assessed at 15 MW in one year, and COD removal energy consumption is 3.6 KWh.Kg^−1^ b COD or 4.9 KWh.m^−3^ ^6^. In our study COD removal was between 74–84% for TWW in *C. variabilis* and *C. sorokiniana* 25% and 40% TWW concentration. Applying microalgae means saving up to 80% in average of the energy consumed for TWW treatment. Adding KWE will further improve the economics of the scale by increasing the removal ratio to 88%. The system can be extra recycled by diluting the new raw TWW with the effluent from the microalgae cultivation process. Adding TWW treatment to our primary objective in saving valuable fresh water and nutrients and producing microalgae feedstock would boost the efficiency of the system. The daily growth parameters such as chlorophyll, sugar content, cell density indicate the growth rate in *C. sorokiniana* and *C. variabilis*, 25% and 40% concentrations, was high during the first 6 days, and most of the progress occurs during this period. It is also reported in other studies on different wastewaters with high organic load that microalgae were able to remove the major ratio of COD, ammonium and phosphorous during the first week^9,26,38^. It represents a major advantage in this system as one cycle of production will consume short time. These conclusions propose that TWW as a medium represents a promising prospect for microalgae cultivation especially when enhanced with KWE.

## Conclusion

In our study cultivating microalgae cells using TWW as a medium viewed a considerable growth and a higher chlorophyll and sugar content compared to control medium in *C. sorokiniana*, *C. variabilis* and *Scenedesmus* sp. Biomass and total lipids were greatly improved in the different concentrations with all strains except in 60% concentration in *Scenedesmus* sp. Water quality parameters, COD, ammonium, and phosphorous witnessed considerable removal ratios. As it balances the nutrients in the medium and stimulate the growth, KWE boosted all growth parameters in all TWW concentrations in *C. sorokiniana*, and reduced the lag phase in high concentrations of TWW, indicating more tolerance to TWW elements load when KWE was added. The application of KWE improved the water quality parameters in effluents. These outcomes prove that TWW represents a good alternative for microalgae cells production, and great improvement could be done by adding different low-cost supplements to optimize the production.
